# Malignant Pleural Effusion Supernatants Are Substitutes for Metastatic Pleural Tumor Tissues in EGFR Mutation Test in Patients with Advanced Lung Adenocarcinoma

**DOI:** 10.1371/journal.pone.0089946

**Published:** 2014-02-28

**Authors:** Dan Liu, Yachao Lu, Zhenli Hu, Ning Wu, Xiaomeng Nie, Yang Xia, Yiping Han, Qiang Li, Guanshan Zhu, Chong Bai

**Affiliations:** 1 Department of Respiratory Medicine, Changhai Hospital, The Second Military Medical University, Shanghai, China; 2 Innovation Center China, AstraZeneca Global R&D, Shanghai, China; National Cancer Center, Japan

## Abstract

**Background:**

Though the possibility of using malignant pleural effusions (MPEs) as alternatives for metastatic pleural tumor tissues (MPTTs) in epidermal growth factor receptor (EGFR) mutation test has been examined, due to the lack of studies comparing the results in matching MPEs and MPTTs, the clinical value of MPEs for advanced adenocarcinoma patients with pleural effusions is not confirmed.

**Methods:**

EGFR mutation statuses in matching MPTTs, MPE supernatants and cell blocks, of 41 patients with advanced lung adenocarcinoma as diagnosed by thoracoscopy were analyzed using amplification refractory mutation system (ARMS).

**Results:**

EGFR mutations were detected in 46.3% (19/41) of MPTTs, 43.9% (18/41) of MPE supernatants and 56.3% (18/32) of MPE cell blocks by ARMS analysis. Generally, the same EGFR statuses were identified in both MPTTs and matching MPE cell blocks of 81.3% patients (26/32), whereas MPTTs and matching MPE supernatants of 87.8% (36/41) patients shared the same EGFR status. Compared with EGFR mutation detection in MPTTs, the sensitivity of EGFR mutation detection in MPE-cell blocks was 87.5% (14/16), specificity was 75.0% (12/16), while the sensitivity of EGFR mutation detection in MPE-supernatants was 84.2% (16/19), specificity was 90.9% (20/22).

**Conclusions:**

The high concordance of EGFR mutation statuses between MPEs and MPTTs in lung adenocarcinoma patients with pleural metastasis as determined by ARMS analysis suggests that MPEs, particularly MPE supernatants, may be substitutes for MPTTs in EGFR mutation test.

## Introduction

Lung cancer is the most frequently diagnosed cancer and the most common cause of cancer-related mortality worldwide.[1], [2] Treatment with epidermal growth factor receptor tyrosine kinase inhibitors (EGFR-TKIs) have significantly improved the prognosis of patients with advanced lung adenocarcinoma, particularly those with EGFR gene mutations.[3], [4]

Currently, tumor tissues obtained by surgery or biopsy (including thoracoscopic pleural metastatic tissue biopsy) are commonly used for EGFR mutation test,[5]–[7] but unfortunately, it is difficult to obtain adequate amount of tumor tissues from patients with advanced lung adenocarcinoma. Some studies have analyzed the EGFR mutation rate in pleural effusions and the relationship between the mutation rate and patient response to gefitinib,[8]–[11] whereas a previous study investigated the EGFR mutation rate in malignant pleural effusions (MPEs) of lung adenocarcinoma and compared it with the mutation rate in surgically resected specimens of lung adenocarcinoma from patients without MPEs.[12] However, to the best of our knowledge, no study has been done to compare EGFR mutation statuses between MPEs and their matching metastatic pleural tumor tissues (MPTTs). Furthermore, the sensitivity and specificity of EGFR gene mutation detection in MPEs remain unknown comparing with that in MPTTs. The purpose of this study is to analyze the EGFR gene mutation rates in MPEs and matching MPTTs obtained by thoracoscopic pleural metastatic tissues biopsy from patients with advanced lung adenocarcinoma and determine if MPEs are good substitutes for MPTTs in EGFR gene mutation test. Meanwhile, the sensitivity and specificity of the mutation tests from MPE supernatants and their matching cell blocks were compared to determine which are of more clinical value.

## Materials and Methods

### Patients and samples

This study was carried out at Changhai Affiliated Hospital of the Second Military Medical University(Shanghai, China)and the procedures were approved by the Institutional Ethics Committee of Changhai Hospital. All patients had signed an informed consent form for the use of these samples in molecular analysis. Patients were eligible for inclusion in the study for further analysis if they met the following criteria:(1)the patients who were highly suspect for lung malignant disease; (2) patients already have pleural effusion at clinic; (3) thoracoscopy was required to make clear the causes of pleural effusion and collect the tumor sample biopsy; (4) metastatic lung cancer were diagnosed by biopsy; (5)Performance status (Eastern Cooperative Oncology Group performance status (ECOG PS)) ≤2. From April 2011 to June 2013, 23 males and 18 females with a median age of 55 years (range, 29 to 78 years), including 13 smokers and 28 non-smokers, were enrolled at the Respiratory Department of Medicine of Changhai Hospital. All patients were pathologically diagnosed as lung adenocarcinoma with pleural metastasis. None of them had received prior EGFR-TKIs therapy. Detailed patient information is listed in Table 1. Paired MPTT and MPE samples were collected for EGFR mutation analysis.

**Table 1:** Patients information.

Characteristics (n = 41)	Patients number	Percentage (%)
Age		
Average	55±12	
Range	29–78	
Sex		
Male	23	56.1
Female	18	43.9
History of Smoking		
Smoking	28	68.3
Non-smoking	13	31.7
Therapy		
None	39	95.1
Chemotherapy	2	4.9
Radiotherapy	0	0
Targeted Therapy	0	0

MPTT samples were obtained through a semi-rigid thoracoscope (LTF-240, Olympus Optical Co Ltd, Tokyo, Japan). MPE samples (250–500 mL) were collected from each patient and centrifuged at 1,000 g for 10 min at room temperature within one hour of collection and all MPE samples were collected before the MPTT procedure to reduce the confounder factors. Ten milliliters of supernatant was stored at −80°C for further analyses. The cell pellets were fixed in 10% neutral-buffered formalin, and then embedded in paraffin to make the MPE cell blocks. Each formalin-fixed, paraffin-embedded (FFPE) sample (MPTT, MPE-cell block) was cut into 5 µm-thick sections that were incubated at 37°C for 3 hr and then stored at room temperature.

All slides from MPTT and MPE-cell blocks were reviewed and diagnosed as lung adenocarcinoma by three different pathologists. All FFPE tissues were pathologically examined to confirm the presence of lung adenocarcinoma and to determine the percentage of tumor cells. Since the sensitivity of ARMS analysis was approximately 1%, only tissue samples with 1% tumor cells or more were selected for the EGFR mutation analysis. Accordingly, all FFPE MPTT samples and 32 of 41 MPE-Cell blocks were qualified for further EGFR mutation analysis.

### Genomic DNA extraction and EGFR mutation analysis

Genomic DNA in FFPE samples (10–12 serial sections) and MPE supernatants were extracted by QIAamp DNA FFPE tissue kits (Qiagen, Hilden, Germany) and QIAamp circulating nucleic acid kits (Qiagen, Hilden, Germany) by following the manufacturer's protocols. The concentration of DNA samples were measured by NanoDrop 2000 Spectrophotometer (Thermo Scientific, Waltham, USA). The DNA was diluted to 2–3 ng/µl to be used in EGFR mutation test.

EGFR mutations were analyzed by using of a Human EGFR Gene Mutations Fluorescence PCR Diagnostic Kit (Amoy Diagnostics, Xiamen, China), which is based on the ARMS technology. The assay can identify the 29 most common types of EGFR mutations currently described in lung cancers. These EGFR mutations included 19 types of deletions in exon 19, 3 types of insertions in exon 20, T790M, L858R, L861Q, G719X and S768I point mutations. All experiments were done by following the manufacturer's protocols. Briefly, 10 ng genomic DNA was added to 45 µL PCR master mix containing PCR buffer, DNA polymerase, PCR primers, fluorescent Taqman probe specific for each individual EGFR mutation. After 47 amplification cycles, the fluorescent signal was collected from FAM and HEX channels.

### Statistical analyses

Χ^2^ test was used for categorical variables. The concordance rate of EGFR mutations and Cohen's kappa coefficients were calculated between MPTTs and MPEs. Cohen's kappa coefficient was calculated as: kappa  =  (Po–Pe)/(1–Pe), where Po is the observed concordance rate and Pe is the expected probability of chance agreement. In general, kappa values of 0.4–0.6 indicate moderate agreement and values more than 0.6 indicate a significant agreement between observations[13]. The Youden's index, the difference between the true positive rate and the false positive rate was also calculated to reflect the reliability of EGFR mutation test, while indexes more than 0.7 indicate significant reliability. A P-value less than 0.05 was considered statistically significant. The statistical analyses were carried out using SPSS 17.0 for Windows (SPSS, Chicago, IL, USA).

## Results

### EGFR mutations in MPTTs

All of the 41 MPTT specimens successfully passed pathological quality control (containing 5%–60% tumor cells). EGFR mutations were found in 46.3% (19/41) of MPTT samples. The most frequent mutations observed were the deletion mutation in exon 19 (12/19, 63.2%) and the point mutation (L858R) in exon 21 (7/19, 36.8%). As listed in Table 2, the rate of EGFR mutation was significantly higher in non-smokers (57.1%, 16/28) than in smokers (23.1%, 3/13) (P = 0.042). Although there was no statistical significance, the mutation rate was higher in women (61.1% (11/18) than in men (34.8%, 8/23) (*P* = 0.093). EGFR mutation status was not correlated with patients' age (*P* = 0.754).

**Table 2:** EGFR mutations in MPTT samples.

	Number of patients	Number of mutations	Mutation frequency (%)	*P*-value
Gender				
Male	23	8	34.8	
Female	18	11	61.1	0.093*
Smoking history				
Former and current smoker	13	3	23.1	
Never smoker	28	16	57.1	0.042**

*: Male versus Female, ** Smoking verse Non-smoking

### EGFR mutations in MPEs

As summarized in Supplementary Data (Table S1), the ARMS analysis showed that EGFR mutations were present in 56.3%% (18/32) of MPE cell blocks. Among these mutations, 10 were exon 19 deletion, 7 were L858R, and 1 was S768I. The frequency of EGFR mutation in the 41 MPE supernatants was 43.9% (18/41). Among these samples, 10 were exon 19 deletions, 7 were L858R, and 1 was S768I. If EGFR mutation was present in either MPE supernatants or MPE cell blocks, the MPE samples was assessed as positive for EGFR mutation. Accordingly, the frequency of EGFR mutations in MPE samples (supernatants and cell blocks) was 53.7% (22/41). Among these mutations, 13 were deletions in exon 19, 8 were L858R, and 1 was point mutation in exon 20 (S768I). MPE cell blocks were generated from MPE samples, and subjected to EGFR mutation analysis accordingly.

### The concordance of EGFR mutations in MPPT and MPE samples

The EGFR mutations in MPTT and MPE samples from the same patients were summarized in Table 3. Generally, the same EGFR statuses were identified in both MPTT samples and MPE cell blocks of 81.3% patients (26/32). Different EGFR mutations were identified in one patient's MPTT sample and MPE cell block, ARMS analysis showed that the MPTT sample harbored an exon 19 deletion, while the MPE cell block had S768I point mutation. Compared to the frequency of EGFR mutations in MPTT samples, the concordance with that in MPE cell blocks was 65.0% (13/20). ARMS analysis also revealed that MPTT samples and MPE supernatants of 87.8% (36/41) patients harbor the same EGFR status. The concordance between the frequency of EGFR mutations in MPTT samples and that in MPE supernatants was 76.2% (16/21). The concordance between EGFR status identified by ARMS analysis in MPTT samples and MPE samples was 85.4% (35/41). Compared to frequency of EGFR mutations in MPTT samples, the concordance of that in MPE samples was 73.9% (17/23). The concordance of the EGFR analysis results of MPE cell blocks and that of MPE supernatants was as high as 84.4% (27/32), indicating the high similarity of EGFR mutation status between these two materials.

**Table 3:** Comparison of EGFR mutation between MPE-cell blocks and matched MPTTs.

		MPTT		
		+	−	Total
**MPE-cell block**	+	14*	4	18
	−	2	12	14
	Total	16	16	32

**MPTT**: metastatic pleural tumor tissue; **MPE**: malignant pleural effusion;

**+**: EGFR positive-mutation; **−**: EGFR negative-mutation;

*: among the 14 patients, one patient showed an exon 19 deletion in MPTT versus S768I mutation in the matched MPE-cell block.

Compared with EGFR mutation detection in MPTT, the sensitivity of EGFR mutation detection in MPE cell blocks was 87.5% (14/16), specificity was 75.0% (12/16), the false positive rate was 25.0% (4/16) and false negative rate was 12.5% (2/16) (Table 3); whereas the sensitivity of EGFR mutation detection in MPE supernatants was 84.2% (16/19), specificity was 90.9% (20/22), the false positive rate was 9.1% (2/22) and false negative rate was 15.8% (3/19) (Table 4). The sensitivity of EGFR mutation detection in combined MPEs was 94.7% (18/19), specificity was 81.8% (18/22), the false positive rate was 18.2% (4/22), and false negative rate was 5.3% (1/19) (Table 5).

**Table 4:** Comparison of EGFR mutation between MPE-supernatant and matched MPTTs.

		MPTT		
		+	−	Total
**MPE-supernatant**	+	16	2	18
	−	3	20	23
	Total	19	22	41

**MPTT**: metastatic pleural tumor tissue; **MPE**: malignant pleural effusion;

**+**: EGFR positive-mutation**; −**: EGFR negative-mutation.

**Table 5:** Comparison of EGFR mutation between MPEs and matched MPTTs.

		MPTT		
		+	−	Total
**MPE**	+	18*	4	22
	−	1	18	19
	Total	19	22	41

**MPTT**: metastatic pleural tumor tissue; **MPE**: malignant pleural effusion;

**+**: EGFR positive-mutation; **−**: EGFR negative-mutation.

*: among the 18 patients, one patient showed an exon 19 deletion in MPTT versus S768I mutation in the matched MPE-cell block.

Statistical analysis showed that the kappa values between EGFR mutations in MPTT samples with that in MPE cell blocks, MPE supernatants and MPE samples were 0.625, 0.749 and 0.765, respectively, and they were statistically significant (*P*<0.001) (Table 6).

**Table 6:** Kappa value and Youden index of EGFR mutations in MPPT and that in MPE samples.

	Kappa value	P-value	Youden index
MPE	0.803	<0.001	0.765
MPE-cell block	0.704	<0.001	0.625
MPE-supernatant	0.794	<0.001	0.749

**MPTT**: metastatic pleural tumor tissue; **MPE**: malignant pleural effusion;

## Discussion

The number of lung cancer patients has been increasing and patients with advanced lung adenocarcinoma account for the majority of lung cancer-related death.[1], [14] EGFR-TKIs are highly effective (71.2%) in the treatment of patients with advanced lung adenocarcinoma with activating mutations in the tyrosine kinase domain of EGFR, but have little or no effect on patients with no activating EGFR mutations.[3] Thus, choosing the right therapeutic approaches is critical to effectively treat patients with advanced lung adenocarcinoma, and decisions are made mainly based on the results of EGFR mutation test.[4], [15] To those lung adenocarcinoma patients with pleural effusions, detection of EGFR mutation statuses in MPTTs obtained by thoracoscopic pleural biopsy provides useful information for choosing the right therapeutic approach. However, not all lung adenocarcinoma patients with pleural effusions are suitable for thoracoscopic pleural biopsy. Furthermore, since the status of EGFR mutation in patients with advanced lung adenocarcinoma may change during the process of therapy, multiple biopsies may be required and this presents a challenge to both the patients and physicians.[16]

The purpose of this study is to find an alternative source of sample that is easy and safe to collect to be used in EGFR mutation analysis. MPE is a common complication in NSCLC and its collection is easier, safer, and more repeatable than the collection of MPTTs.[17], [18] Although many studies have been done to show that MPEs are good for detection of EGFR mutations,[9], [12], [19], [20] the MPEs were not matched with corresponding MPTTs, which compromised the credibility of the results. To convincingly demonstrate that MPEs are good alternative specimens for EGFR mutation test, we need to compare the mutation statuses in matched MPEs and MPTTs and show that they are consistent.

Our results showed an 85.4% concordance rate of EGFR mutation between MPEs and matched MPTTs with the kappa coefficients of 0.803, indicating that the status of EGFR mutation in MPEs reflects that in corresponding MPTTs and suggesting that MPEs could substitute for MPTTs to be used in EGFR mutation test. Two possible reasons might have contributed to the difference in the samples that did not show concordance: (1) Even with ARMS technology, the poor quality DNA extracted from samples or low rate EGFR mutation which was beyond the sensitivity limit of the detection might have resulted in false negative; and (2), The tumor was genetically heterogeneous, which means that mutation-positive cells and mutation-negative cells co-existed,[16], [21] and/or in a mixed adenocarcinoma lung nodule,[22] different EGFR mutations could be demonstrated in various parts of the tumor.

It has been found that the EGFR mutation rate in MPEs (53.7%) was higher than that in MPTTs (46.3%). We speculated that this was due to the existence of tumor cells dropped off from MPTTs and cell-free DNAs from broken tumor cells. Nevertheless, this result suggested that MPEs could be more representative specimens in adenocarcinoma patients with pleural metastasis for the EGFR mutation test. Based on this result, we concluded that the EGFR mutation test sensitivity could be improved if both the supernatant and cell blocks of MPEs are used, but this would increase the complexity and cost of the test. To solve this problem, we compared the concordance of the EGFR mutation test results between MPE supernatants and MPTTs, and MPE cell blocks and MPTTs, respectively, and found that in the 41 pairs of MPE supernatants and cell blocks, the supernatants had higher concordance with MPTTs in terms of EGFR mutations. Furthermore, the Youden Index of the tests using the supernatants (0.749) was significantly higher than that of the tests using MPE cell blocks (0.625), suggesting that the results from supernatants could better reflect the real status of EGFR mutation in MPTTs. Herein, we concluded that compared with MPE cell blocks, MPE supernatants are better substitutes for MPTTs in EGFR mutation test and have the following advantages: (1) Their EGFR mutation status is highly concordant with that of MPTTs (87.8% in our studies) with the specificity of 90.9%, false negative rate of 9.1% and Youden index as high as 0.749); (2) By eliminating the need of embedding, the loss of specimen is minimized and the test procedure is simplified; (3) Unlike malignant cancer tissues, DNA in malignant pleural effusions is primarily derived from tumor cells, as long as it is accessible, it is qualified for EGFR test; for malignant cancer tissues, only tissues with a minimum of 1% malignant cancer cells are qualified for EGFR test, since the sensitivity of ARMS analysis was approximately 1%; (4) It is easier to purify DNA from MPE supernatants than from MPTTs.

## Conclusions

In this study, we have compared the EGFR mutation statuses in MPEs and their matching MPTTs from patients who have been diagnosed to have advanced lung adenocarcinoma by thoracoscopic pleural biopsy and demonstrated high concordance rate of EGFR mutations between MPEs and MPTTs as determined by the ARMS analysis. We also found that compared with MPE cell blocks, MPE supernatants showed higher concordance with MPTTs in EGFR mutations status. This result suggests that MPEs, particularly MPE supernatants, may be used as substitutes for MPTTs in EGFR mutation analysis. This information will benefit the treatment of advanced NSCLC patients in determining whether EGFR-TKIs are right to them.

## Supporting Information

Table S1 **Summary of EGFR mutations in MPTT and MPE of same patients.**
(DOCX)
