# Supplementary material for: Malignant Pleural Effusion Supernatants Are Substitutes for Metastatic Pleural Tumor Tissues in EGFR Mutation Test in Patients with Advanced Lung Adenocarcinoma
Source: PLoS One. 2014 Feb 28;9(2):e89946. doi: 10.1371/journal.pone.0089946 (PMC3938554; doi:10.1371/journal.pone.0089946)
Supplement: Table S1 — Summary of EGFR mutations in MPTT and MPE of same patients. (DOCX) [file pone.0089946.s001.docx]

Table S1. Summary of EGFR mutations in MPTT and MPE of same patients

|  | **EGFR mutation status** | | | |
| --- | --- | --- | --- | --- |
| **Serial no.** | MPTT | MPE-CB | MPE-S | MPE* |
| **1** | N | N | N | N |
| **2** | N | L858R | L858R | L858R |
| **3** | 19-del | S768I | N | S768I |
| **4** | 19-del | 19-del | 19-del | 19-del |
| **5** | 19-del | 19-del | 19-del | 19-del |
| **6** | N | 19-del | N | 19-del |
| **7** | 19-del | N | 19-del | 19-del |
| **8** | N | -a | N | N |
| **9** | N | -a | N | N |
| **10** | N | N | N | N |
| **11** | N | N | N | N |
| **12** | N | -a | N | N |
| **13** | 19-del | 19-del | 19-del | 19-del |
| **14** | L858R | -a | L858R | L858R |
| **15** | N | N | N | N |
| **16** | L858R | L858R | L858R | L858R |
| **17** | N | N | N | N |
| **18** | N | -a | N | N |
| **19** | 19-del | 19-del | 19-del | 19-del |
| **20** | 19-del | 19-del | N | 19-del |
| **21** | 19-del | 19-del | 19-del | 19-del |
| **22** | N | -a | N | N |
| **23** | L858R | L858R | L858R | L858R |
| **24** | 19-del | -a | 19-del | 19-del |
| **25** | N | -a | N | N |
| **26** | N | N | N | N |
| **27** | 19-del | 19-del | 19-del | 19-del |
| **28** | 19-del | -a | 19-del | 19-del |
| **29** | N | N | N | N |
| **30** | L858R | L858R | L858R | L858R |
| **31** | N | 19-del | 19-del | 19-del |
| **32** | L858R | L858R | L858R | L858R |
| **33** | L858R | N | N | N |
| **34** | N | L858R | N | L858R |
| **35** | N | N | N | N |
| **36** | N | N | N | N |
| **37** | N | N | N | N |
| **38** | 19-del | 19-del | 19-del | 19-del |
| **39** | 19-del | 19-del | 19-del | 19-del |
| **40** | N | N | N | N |
| **41** | N | N | N | N |

**MPTT**: metastatic pleural tumor tissue; **MPE**: Malignant pleural effusion; **MPE-CB:** MPE-cell block**; MPE-S:** MPE-supernatant**;**

**19-del:** deletion mutations in exon 19; **L858R:** missense mutations in exon 21;

**N:**EGFR negative-mutation; **a:** less than 1% tumor cell content;

*The MPE sample was assessed as positive for EGFR mutation, if EGFR mutation was present in either MPE-supernatants or MPE-cell blocks.
